# Supplementary material for: Healthy-Canteen Displays: A Tactic to Encourage Community Sport Canteens to Provide Healthier Food and Beverage Options
Source: Int J Environ Res Public Health. 2021 Sep 28;18(19):10194. doi: 10.3390/ijerph181910194 (PMC8507705; doi:10.3390/ijerph181910194)
Supplement: Supplementary file 1 [file ijerph-18-10194-s001.zip › ijerph-1381932-supplementary.pdf]

Survey 1

Please select which option best represents the canteen at your venue:

No canteen
Privately owned and operated
Club owned and operated
Council owned and operated
Other (please specify)

Do you think that the canteen as it currently operates has appropriate food/beverage options?

Yes
No

Do you have any influence (given your position at the facility) over products available in the canteen?

Yes
No

How important do you think it is to have healthy food/drink options at the canteen? (with 0 being not i

Have you heard of the food traffic light system before?

Yes
No

How confident are you that you could easily distinguish between a "green" healthy, "amber" choose ca

What are the main barriers to introducing healthier options at your canteen? (Please choose all that ap

People don't want to buy healthy options:
Food waste - fresh food if not bought has to be thrown out:
No time:
Negative financial implication on my total sales:
I have no idea which products can be a healthier alternative to my existing stock:
People playing sport need to have access to sport drinks:
I don't know enough about nutrition to even think about implementing alternative options in the canteen
The facility is locked into options available through a contracted supplier:
I don't know where to source the supply of healthier options:
The price of healthier options is unlikely to be attractive to consumers at our facility:
There is a lack of storage (fridge or dry) for alternative canteen options:
Staff/volunteers would need to be upskilled in food safety practices if healthier options were implemente
Other (please specify):

What impact do you think a healthier canteen will have on sales?

Increase
Reduce
No impact

Survey 2

Please select which option best represents the canteen at your venue:

No canteen
Privately owned and operated
Club owned and operated
Council owned and operated
Other (please specify)

Do you think that the canteen as it currently operates has appropriate food/beverage option

Yes
No

Do you have any influence (given your position at the facility) over products available in the

Yes
No

How important do you think it is to have healthy food/drink options at the canteen? (with 0

Have you heard of the food traffic light system before?

Yes
No

How confident are you that you could easily distinguish between a "green" healthy, "amber"

What are the main barriers to introducing healthier options at your canteen? (Please choose

People don't want to buy healthy options:
Food waste - fresh food if not bought has to be thrown out:
No time:
Negative financial implication on my total sales:
I have no idea which products can be a healthier alternative to my existing stock:
People playing sport need to have access to sport drinks:
I don't know enough about nutrition to even think about implementing alternative options in t
The facility is locked into options available through a contracted supplier:
I don't know where to source the supply of healthier options:
The price of healthier options is unlikely to be attractive to consumers at our facility:
There is a lack of storage (fridge or dry) for alternative canteen options:
Staff/volunteers would need to be upskilled in food safety practices if healthier options were i
Other (please specify):

What impact do you think a healthier canteen will have on sales?

Increase
Reduce
No impact

Survey 3

Please select which option best represents the canteen at your venue:

No canteen
Privately owned and operated
Club owned and operated
Council owned and operated
Other (please specify)

Do you have any influence (given your position at the facility) over products available in the c

Yes
No

Did you visit the healthy canteen display at the Basketball Managers Convention and Trade S

Yes
No

Have you made any changes as a result of visiting the healthy canteen display?

Yes
No

Were the changes made in relation to   (Please select all that apply):

Product supply and availability (e g introduced new products):
Pricing of products (e g increased the price of ‘red’ or unhealthy options):
The availability of products visible to consumers (e g reduced or removed ‘red’ or unhealthy op
Marketing and promotion (e g posters or signage):
Other (please specify):

Do you have any plans to make changes as a result of visiting the healthy canteen display?

Yes
No

What changes are you interesting in making? (Please select all that apply)

Product supply and availability (e g introduced new products):
Pricing of products (e g increased the price of ‘red’ or unhealthy options):
The availability of products visible to consumers (e g reduced or removed ‘red’ or unhealthy op
Marketing and promotion (e g posters or signage):
Other (please specify):
